# Patient and provider perspectives on reducing risk of harm in primary health care: a qualitative questionnaire study in Sweden

**DOI:** 10.1080/02813432.2020.1717095

**Published:** 2020-01-24

**Authors:** Rita Fernholm, Martin J. Holzmann, Karolina Malm-Willadsen, Karin Pukk Härenstam, Axel C. Carlsson, Gunnar H. Nilsson, Caroline Wachtler

**Affiliations:** a Division of Family Medicine and Primary Care, Department of Neurobiology, Care Sciences and Society, Karolinska Institutet, Huddinge, Sweden;; b Department of Medicine, Karolinska Institutet, Stockholm, Sweden;; c Functional Area of Emergency Medicine, Karolinska University Hospital, Stockholm, Sweden;; d Stockholm Region, Sweden;; e Department of Learning, Informatics, Management and Ethics, Medical Management Centre, Karolinska Institutet, Stockholm, Sweden

**Keywords:** Patient safety, primary health care, medical errors, continuity of patient care, risk assessment, quality of health care

## Abstract

**Objective:** To explore how patients, that had experienced harm in primary care, and how primary providers and practice managers understood reasons for harm and possibilities to reduce risk of harm.

**Design:** Inductive qualitative analysis of structured questionnaires with free text answers.

**Setting:** Primary health care in Sweden.

**Patients/subjects:** Patients (*n* = 22) who had experienced preventable harm in primary health care, and primary care providers and practice managers, including 15 physicians, 20 nurses and 24 practice managers.

**Main outcome measures:** Categories and overarching themes from the qualitative analysis.

**Results:** The three categories identified as important for safety were continuity of care, communication and competence. With flaws in these, risks were thought to be greater and if these were strengthened the risks could be reduced. The overarching theme for the patient was the experience of being neglected, like not having been properly examined. The overarching theme for primary care providers and practice managers was lack of continuity of care.

**Conclusion:** Primary care providers, practice managers and patients understood the risks and how to reduce the risks of patient safety problems as related to three main categories: continuity of care, communication and competence. Future work towards a safer primary health care could therefore benefit from focusing on these areas.

**Key points**
Current awareness:
  • Patients and primary care providers are rather untapped sources of knowledge regarding patient safety in primary health care.
Main statements:
  • Patients understood the risk of harm as stemming from that they were not properly examined.
  • Primary care providers understood the risk of harm to a great extent as stemming from poor continuity of care.
  • Patients, primary care providers and practice managers believed continuity, communication and competence play an important role in reducing risks.

## Introduction

Patients experiencing harm in healthcare is a worldwide problem [1]. According to a systematic review in 2012, medication errors, central line infections and hospital-stay related venous thromboembolism are the most common types of harm in healthcare overall [2], and procedure-related injuries [3,4] are additionally common in hospitals.

Preventable harm to patients in primary health care (PHC), is an issue of rising importance since it represents the largest volume of encounters [5] and in this setting the types of harm differ to some extent from that of the hospitals [6]. In PHC, diagnostic error — defined as delayed, missed or incorrect diagnoses [7] — is a common type of preventable harm, especially among serious harm [7–9]. Diagnostic error can, for example, be due to misinterpretation of symptoms and signs or may stem from breakdown in communication, resulting in patients not receiving correct treatment in a timely manner. The frequency of diagnostic errors in outpatient care has been estimated at 5% [6,10].

Medication error, defined as errors in prescribing, dispensing or administering medication with the result that the patient fails to receive the correct drug or the indicated proper drug dosage, occurs at a rate of 3 to 10% [11] and even higher in patients with polypharmacy (56%) [12], and also contribute to preventable harm in PHC.

In 2015, the LINNEUS collaboration published a series of articles concerning patient safety in PHC [13–19]. The four main areas of safety were diagnostics, medication, communication and organisation [13], and they emphasised the importance of involving patients in improving safety [15,19]. The views and experiences of patients and primary care providers (PCPs), which here include physicians and nurses, regarding patient safety risks and how to reduce them, are only partially understood. In a systematic review in 2015, patients identified medication errors, communication and coordination of care as the most prevalent problems of care [20]. In the UK, 113 PHC clinicians were asked *via* questionnaire to identify problems and solutions to patient safety issues. Poor communication between secondary and primary care was emphasised and standardised discharged summaries were suggested [21,22]. In addition, general practitioner (GP) perspectives on patient safety incidents have been explored using critical incident technique interviews of 30 Irish GPs in which diagnostic and medication errors were the most described, and breakdown in communication was seen as a common contributory factor [23].

However, to our knowledge, no study has investigated both the opinions of patients who were harmed in PHC and the opinions of PCPs and practice managers regarding the most important patient safety risks and how to reduce those risks.

The aim of this study was to explore how patients harmed in PHC, PCPs and practice managers understood reasons for harm and possibilities to reduce risk of the two major types of harm in PHC, diagnostic and medication errors.

## Material and methods

An inductive qualitative analysis of free-text answers to structured questionnaires about safety risks and how to reduce them. The context was Swedish primary health care.

### Questionnaire construction

Based on the experiences from Tudor et al. [21,22], we developed two separate questionnaires with open-ended questions aiming to explore different perspectives of patients as well as providers. The patient questionnaire was designed to capture the perspective of patients who had experienced diagnostic or medication errors on why the error had occurred, and how they thought similar errors could be prevented.

The provider questionnaire included questions based on WHO-identified areas of patient safety issues [24], with the intention of identifying what PCP and practice managers saw as reasons for diagnostic errors and medication errors in the identified areas and how they thought those risks could be reduced. There were also general open-ended questions about risks of diagnostic errors and medication errors and how risk of patient harm could be reduced in general. Both questionnaires were translated to Swedish and then validated by back-translation. The questionnaires are presented in Supplementary material 1.

#### Data collection

A total of 22 individuals with personal experience of medication or diagnostic error were recruited with purposive sampling. The patients were chosen from Landstingens Ömsesidiga Försäkringsbolag (LÖF), a nationwide non-punitive malpractice carrier and insurance company. The individuals had received compensation for preventable medication or diagnostic error during the previous seven years (2010–2017). Contact information for 20 individuals from diagnostic errors and 20 from medication errors was provided by the LÖF registry.

Letters were sent to all potential participants with information about the study and the questionnaire which could be answered on paper (eight responses) or online (one response). Since we anticipated that patients might perceive the questionnaire as sensitive and difficult to answer we decided to contact respondents by phone in order to be able to overcome language barriers and to provide a verbal alternative to answering the questionnaire. Thirteen respondents answered the questionnaire in this manner. The answers were written down verbatim. In total, information from eight patients who had received compensation for medication and 14 who had received compensation for diagnostic errors were included.

It was not possible to access the identity of the PCPs that had been involved with the patients included in the study, so the harm that patients had experienced was not in contact with the PCPs included in the study. A total number of 59 PCPs and practice managers were recruited from the Stockholm region, 15 physicians, 20 nurses and 24 practice managers. The practice managers were to 93% clinicians by training. The recruitment was made by snowballing, first sending e-mails to all the practice managers (*n* = 195) in the Stockholm Region asking if they would answer the questionnaire and requesting the email addresses of physicians and nurses in their practice. All questionnaires were administered online.

The electronic responses were saved in a password protected database and written material was stored in locked storage, to which only author RF had a key. The texts were anonymised and assigned coded labels so the type of respondent (doctor, nurse, etc.) and the question asked could be identified.

#### Qualitative analysis

An inductive content analysis approach as described by Elo and Kygnas [25] was used. All material is first read through and then the entire dataset is coded in a process where the researcher identifies meaningful units of text and assigns a code to each unit. Codes are short words or phrases, derived from the dataset, reflecting the central concept of the meaningful unit, and are used as a ‘heuristic device’ in order to better understand the content of a qualitative dataset [26]. Similar codes are then grouped together into subcategories, and subcategories are organised into categories, thereby abstracting the many details of the dataset to a higher logical level [27]. Finally, two themes, one for the patients and one for the PCPs and practice managers, were identified that ran through all the categories. The authors RF and KMW did a first complete read through. KMW did the initial coding of the total dataset and RF coded a sample of approximately 30% and the researchers compared and discussed coding frameworks until consensus was reached. Initial categorisation was the result of a consensus discussion between RF and KWM. Thereafter, author CW read all text and coded approximately 10% of the text. CW, RF and KWM finalised the subcategories and organised these into categories in a consensus discussion. Findings are reported descriptively. The codes for reasons for harm/experienced risks and ways to reduce risk were merged in categories important for safety. There were no obvious differences in codes or subcategories between physicians, nurses and practice managers, so these groups are presented together as PCPs and practice managers.

## Results

Twenty-two patients from different parts of Sweden responded to the questionnaire. Fifty-nine PCP and practice managers from the Stockholm region in Sweden responded to the questionnaire, with the content from different groups similar to each other and therefore were analysed as one group.

**Figure 1.**
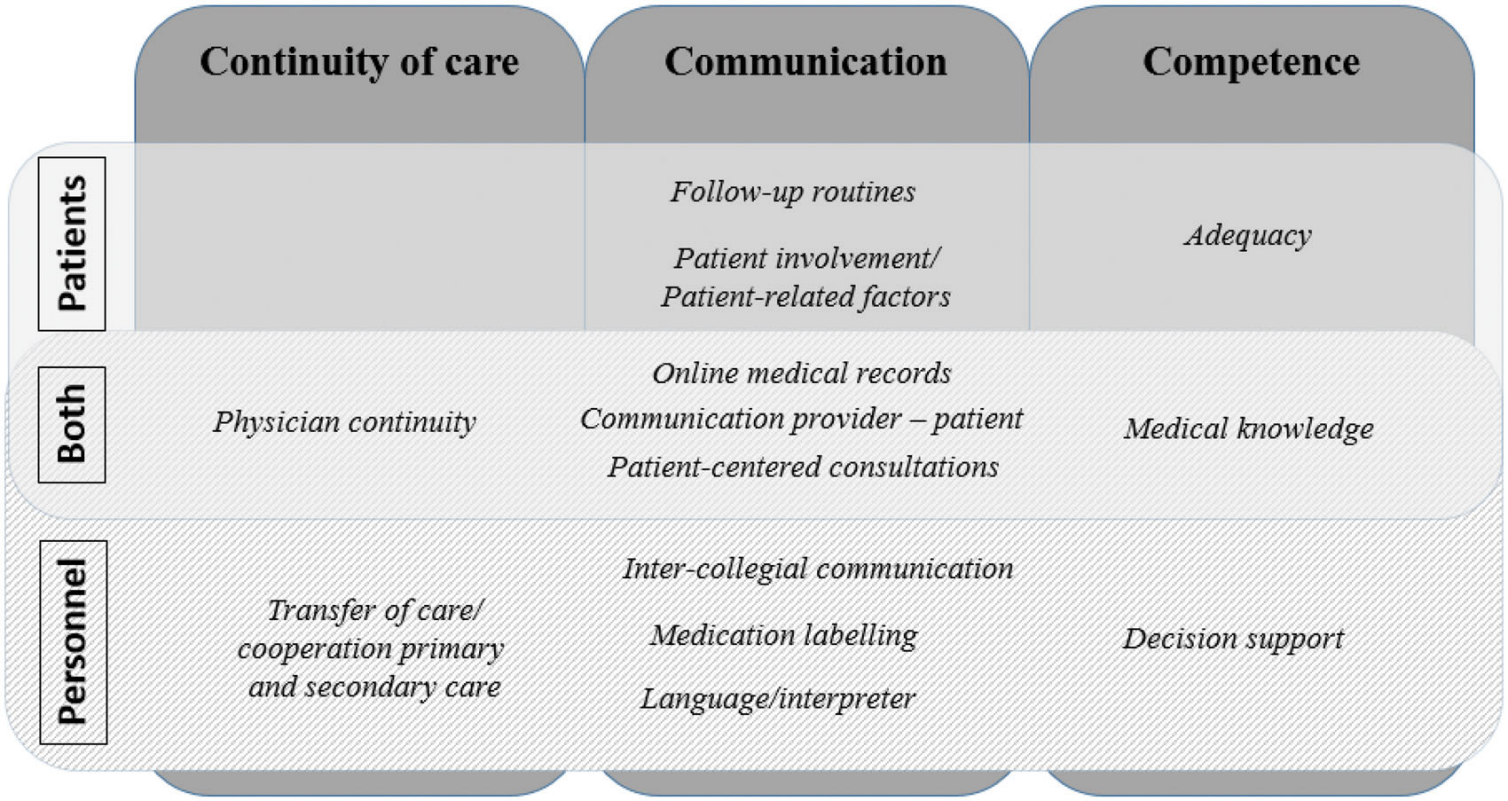
Categories (continuity of care, communication and competence) and subcategories important for safety in primary health care, derived from qualitative analysis of patients, PCP and practice managers. With flaws in these, risks were thought to be greater and if these were strengthened the risks could be reduced (2010–2017).

Three categories were experienced as central to patient safety and the possibilities to reduce risks: ‘continuity of care, communication and competence’, the later including skills, capacity and qualification. In total 13 subcategories were identified from the material, eight from the patients and 10 from the PCP and practice managers, with five subcategories shared (Figure 1).

Quotes are referred to as P1 for patient 1, D1 for doctor 1, N1 for nurse 1 and M1 for practice manager 1 and, so on.

### Continuity of care – patients

In the category continuity of care patients identified ‘physician continuity’ as important. Physician continuity meant to see the same doctor repeatedly. The patients had a vague sense of risk when care was experienced as fragmented. The patients also expressed the discomfort of seeing new doctors and having to tell their story all over again.

‘It is frustrating to meet many different doctors’. (P6)

### Communication – patients

Patients draw attention to problems concerning ‘follow-up routines’, both follow-up of the physical examination and information about what to do if symptoms did not resolve, as well as follow-up of medication changes. Patients felt they were left with the responsibility to make sure that the follow-up took place.

‘Bad follow-up. I had to call back myself and then a nurse made assessment over the phone, no visit’. (P7)

Patients also described ‘low patient involvement and patient related factors’ as an understanding of risks. These could lead to delay in seeking care, resulting in a delayed diagnosis.

‘In addition, I have a high threshold for pain resulting in that I did not seek health care right away…. That could have been to my disadvantage’. (P5)

‘Online medical records’ accessible by everyone engaged in the patient’s care was seen by patients as important.

‘The pharmacy and the doctor should of course have the same medical record’. (P11)

Patients valued ‘patient-centred consultations’ and understood that the lack thereof poses a risk of preventable harm to the patient.

‘The doctor needs to listen to the patient and read the patient’s history. The doctor needs to listen to and understand the patient’. (P8)

### Competence – patients

Patients experienced poor ‘adequacy’ as a risk for patient safety, including errors of commission and errors of omission, for example not feeling that they had been examined properly. Patients described getting the wrong dose of a medication:

‘The doctor was careless and read the medication record incorrectly’. (P11)

The patients felt that the doctor did not listen to them, did not know what to do and that a major risk constituted of that the doctor did not examine them in the right way or not at all.

‘I was not taken seriously, and I was not properly examined’. (P12)

‘The visit was fast, like an assembly line’. (P6)

The patients experienced that the doctors lacked competence. It is not known if the doctors did have the competence but failed in communicating it or if there were an actual lack of it.

‘I experienced harm because of lack of knowledge, effect of stigmatization and [the doctor] not listening to my symptoms’. (P1)

‘Medical knowledge’ was addressed and the patients liked the idea of a second opinion.

‘[There is] a lower risk of missed diagnosis with a second opinion’. (P3)

### Continuity of care – PCPs and practice managers

In the category continuity of care, PCP and practice managers identified ‘physician continuity’ as important. The PCPs and practice managers understood the risk of gaps in care in relation to be able to conclude the correct diagnosis and make sure the medication is adequate.

‘If the doctor knows his or her patients, and the patient can meet the same doctor every visit, the likelihood that the doctor will notice when something isn’t right increases’. (D13)

‘Poor continuity of care results in that no one takes responsibility for follow-up’. (M8)

PCPs also drew attention to the risk with ‘transfer of care’. Of particular concern were changes to medication regimens during in-patient care or contact with another specialty. There were also suggestions of solutions, for example:

‘When discharging a patient from the hospital to PHC, oral report via phone should be mandatory the same day as the patient is discharged’. (D15)

### Communication – PCPs and practice managers

In the category communication, ‘online medical records’ accessible by everyone engaged in the patient’s care were seen by PCPs and practice managers as critical to patient safety. They saw the risk when information about the patient’s medication was scattered between systems. A national medication list was considered as one of the primary ways to avoid medication error:

‘We need to prioritize implementation of a national medication prescription module so every change in medication is continually updated in every electronic medical record system’. (M1)

Good ‘communication between the PCP and the patient’ was stressed as important. The doctors felt that they needed to use ‘patient-centred consultation’ techniques to prevent error. Patient-centred consultations were seen based on ‘open questions’ (D15) and:

‘We need to change our perspective and see the patient as a co-creator of their care, based on his or her needs, expectations and resources’. (D7)

The PCPs particularly emphasized ‘inter-collegial communication’ in the form of teamwork as important for patient safety. Teamwork can be difficult in primary care where PCPs often work in different rooms. They wanted arenas for discussion.

‘We want more time for discussion between doctors and nurses’. (N9)

‘We need to work more in teams to strengthen the “safety net” [the ability to detect and mitigate risks], then we can increase our chances of catching misunderstandings’. (D12)

‘Medication labelling’ of the drug packaging was seen a risk for medication error identified by PCPs and practice managers. They understood the risk that the patients receive the wrong medication or that the patient took double or triple of a medication because the name differed. Generic prescribing or better labelling were viewed as possible improvements:

‘If our patients, particularly older patients, are going to have a chance to manage their medications themselves, the medication at the pharmacy should be labelled better, so that the patient knows what medication he or she takes’. (N9)

‘Language/interpreter’ was a category identified by PCP and practice managers as a risk for patient safety in regard to both medication and diagnostic errors. For example, one practice manager suggested that:

‘Information needs to be available in the patients language and should be delivered by an interpreter at the visit’. (M2)

### Competence – PCPs and practice managers

In the category competence, ‘medical knowledge’ was addressed. Medical knowledge includes the knowledge of correct work-up or investigation of symptoms, diagnoses and treatment and the use of that knowledge. The PCPs saw that the lack of knowledge in other PCPs posed a risk of errors. The PCPs did not express that they believed they lacked knowledge themselves, even though they saw the benefit of using second opinion.

‘Sometimes with a second opinion can be good if the doctor does not know how to proceed’. (D8)

PCPs and practice managers saw ‘decision support’ as way to mitigate risk for patient safety. Guidelines and checklists were also seen as useful:

‘…support in form of guidelines on line are absolutely helpful’. (M8)

#### Overarching themes

The patients pointed out the importance of the meeting with the care provider. A recurring phenomenon in the material was the experience of not being listened to, not examined properly or not examined at all. Being neglected was identified as an overarching theme for the patients.

The PCP and practice managers emphasised the importance of continuity of care, an overarching theme for this group. Lack of continuity was also seen as a risk while good continuity was seen to mitigate other risks, like the risks involved in transfer of care, the lack of online medical records, or ambiguous medication labelling. Continuity can strengthen patient-centred consultations and complement knowledge by the trust in the doctor-patient relationship, so that the patient comes back when their symptoms persists.

Stress/lack of time was mentioned by both patients and providers as a safety risk within all three categories.

## Discussion

### Main findings

Continuity of care, communication and competence were found to be important to patient safety by patients, PCPs and practice managers. Patients felt neglected while PCPs and practice managers felt the need for better continuity of care.

### Strengths and limitations

A strength of the study is that it included the perspective of patients that have experienced preventable harm in contact with PHC as well as the perspective of PCPs and practice managers.

The study captures the perspective of 22 patients that had experienced harm and there might therefore be perspectives that were not elucidated. These patients have all reported harm on their own initiative, making them a subpopulation that might be more dissatisfied with care given than other patients that have suffered from harm. On the other hand, they might have higher motivation to share thoughts of how health care could be improved.

We hypothesised that patients would find it easier to approach the questions if the questions were related to their personal experience of harm. The answers were thus limited to their unique experience. However, we did see saturation in the responses collected.

It was a methodological weakness that we used two different methods of data collection for the patients, filling out a questionnaire themselves or answering to the questionnaire verbally over the phone. The verbal responses were written down verbatim, thus making the clarity of the data comparable to the written responses. The two different mechanisms for collection responses could affect the richness of data and might represent different subgroups of the respondents. The respondents who choose to answer the questionnaire in written form provided rich responses while the verbal responses where somewhat more concise which might be an effect of that they were collected over the phone. There was no difference in the perspective between the two groups.

We share the experience reported by Tudor et al. [21,22] about the difficulty in capturing the patients perspective. However, the choice to develop two separate questionnaires and initiate personal contact with patients enabled us to include the patient perspective as well as the perspective of providers.

The PCPs were not selected from a population of persons with experience of preventable harm per se, since that was not practically possible, but from the fact that they were willing to fill out the questionnaire. This might mean that the respondents that chose to answer the questionnaire represent a subgroup of providers more interested inpatient safety.

In the provider questionnaire, a limitation was that the information collected to some extent was guided by the WHO areas of safety risks. However, the responses yielded many other perspectives of risks as well. Qualitative data collected by written responses to questionnaires can be less rich, compared to other methods such as interviews or focus group. However, the questionnaire allowed us to collect insights on this unexplored area from a larger group. In this study, the physicians, nurses and practice managers were analysed together which might hide the subtler differences in perspectives between professions. Differences in perspectives between doctors and nurses on patient safety has been shown in earlier studies [28]. In addition to physician perspectives in previous studies of Tudor et al. [21,22], the views of nurses, practice managers and patients provided some additional perspectives.

In the reporting of results, transferability is maximised by clearly defining the context, the recruitment process, and the analytic process [27]. Additionally, there were multiple researchers involved in data analysis and discussion, and the findings are reported with appropriate example quotations, also allowing for transferability [27].

### Comparison with previous studies

Patients emphasised lack of continuity, poor communication and poor quality of the patient -provider encounter as risks. Most importantly, the patients felt neglected during the patient-provider encounter, that they were not listened to and that they should have been more thoroughly examined. This is an interesting phenomenon not researched before to any greater extent. Other studies from PHC have shown that safety climate and openness of communication had the largest potential for improvement [28]. Maybe PCPs did not communicate why certain examinations, diagnostic tests or imaging were not made thus failing to create a shared understanding of the situation. Patients also experienced lack of medical knowledge as a major risk while the PCPs answering the questionnaire did not emphasised that aspect. It is possible that the experienced lack of knowledge reflects poor communication: that the clinician has the knowledge but does not communicate that to the patient. PCPs did identify lack in knowledge in other PCPs as a potential problem, but not that they lacked knowledge themselves. This might reflect the ‘blind spot bias’ [29,30] – it is harder to see your own shortcomings.

The PCPs understood many of the risks as caused by gaps in the continuity of care. Tudor et al. [21,22] also identified continuity issues such as poor communication between primary and secondary care and suggested standardised discharge summaries and more rigorous systems for follow-up of abnormal test results. Lack of continuity of care in PHC has been raised as a serious threat to safe care [31] and good continuity is shown to result in fewer hospital admissions [32,33] and even lower mortality [34]. Patients also pointed out the importance of physician continuity.

### Implications for health care and research

Sweden, as other countries, is adjusting to deliver care closer to the patient and preferably outside the traditional hospital setting. The initiative is called ‘Coordinated development for good quality, local health care’ [35] and will probably transform health care delivery in Sweden. Resources will need to be redirected to PHC to enable safe care in that new setting.

Based on the current study health care could benefit from working on the quality of the provider-patient encounter and to improve continuity of care. PCPs need to develop ways to identify and address the patients experience of lack of competence of the PCP and make sure that the patient feels, and of course is properly examined. Practice managers should have a strategy to optimise continuity, especially for older patients with higher risk of preventable harm [36,37]. The similarities and differences of perspectives found in the study could be further explored in order to provide a base for development and implementation of patient safety interventions.

The information from this study will be used to create a larger survey were the respondents will rank different risks and solutions to safety issues in PHC. The results of that survey can then be used for identifying areas of action.

## Conclusion

In conclusion, PCPs and practice managers, as well as patients understood the risks and how to reduce the risks of patient safety problems as related to three main categories: continuity of care, communication and competence. Implications for work towards a safer PHC could therefore benefit from focusing on these areas. In addition, the inclusion of the perspectives of patients, primary care providers and practice managers could increase the possibility of developing relevant countermeasures for safety risks in PHC.

## Data Availability

The datasets used and/or analysed during the current study are available from the corresponding author on reasonable request.
